# Coronavirus Disease 2019-Induced Rhabdomyolysis

**DOI:** 10.7759/cureus.10123

**Published:** 2020-08-29

**Authors:** Sreenath Meegada, Vijayadershan Muppidi, Donald C Wilkinson, Suman Siddamreddy, Shravan K Katta

**Affiliations:** 1 Internal Medicine, University of Texas Health Science Center/Christus Good Shepherd Medical Center, Longview, USA; 2 Internal Medicine, Indiana University Health, Indianapolis, USA; 3 Internal Medicine, Christus Good Shepherd Medical Center, Longview, USA; 4 Internal Medicine, Baptist Health Medical Center, Little Rock, USA; 5 Internal Medicine, University of Texas, Arlington, USA; 6 Internal Medicine, Texas Health Arlington Memorial Hospital, Arlington, USA

**Keywords:** covid 19, rhabdomyolysis, elevated ck levels, corona virus, muscle aches, fever, high fever

## Abstract

Rhabdomyolysis is caused by necrosis of muscles and leakage of intracellular contents into blood circulation. It is most commonly caused by trauma, crush injuries, drugs, toxins, immobilization, compartment syndrome, prolonged surgical procedures, and less likely by infections. Infection-related rhabdomyolysis is rare, but not uncommon, and is seen in both viral and bacterial infections. Extrapulmonary manifestations of coronavirus disease 2019 (COVID-19) include thrombotic like pulmonary emboli, acute cerebrovascular accident, myocardial infarction, cardiac arrhythmias, liver injury, gangrene, diarrhea, acute renal failure, and so on. We here describe a case of COVID-19-induced rhabdomyolysis in a 19-year-old Hispanic male presenting with muscle aches, fatigue, fevers, and no pulmonary symptoms.

## Introduction

Coronavirus disease 2019 (COVID-19) is caused by the severe acute respiratory syndrome coronavirus 2 (SARS-CoV-2) virus, a type of coronavirus. It started in Wuhan, China and in a matter of three months, spread worldwide and was declared a global pandemic by the World Health Organization (WHO) [1]. According to the Johns Hopkins University COVID-19 resource center, as of August 16, 2020, more than 21.5 million cases and 773,000 deaths have been reported worldwide [2]. According to the Centers for Disease Control (CDC), the most common symptoms of COVID-19 are fever, chills, cough, sore throat, shortness of breath, chest pain, myalgias, and anosmia. It is widely believed to be a respiratory illness. However, some atypical presentations and extrapulmonary manifestations have been reported [3-5]. Rhabdomyolysis has been reported in COVID-19. So far, there have been a few case reports of COVID-19 with rhabdomyolysis. It is still a rare presentation but potentially life threatening. Here, we present a case of a young male with COVID-19 and rhabdomyolysis. We discuss the presentation and management aspects to increase the awareness of this association among the medical community.

## Case presentation

A 19-year-old Latino male with past medical history significant for ulcerative colitis and non-adherence to prescribed mesalamine presented to the emergency department with a complaint of three days of diffuse abdominal pain with the most severe pain in the left upper quadrant. The patient endorsed headache and severe back pain. Two weeks prior to presentation, the patient fell and sustained an abrasion on his left knee which over the course of the next few days became erythematous, edematous, and febrile. One week prior to presentation, he went on vacation to Colorado where he saw a physician for his knee wound and pain and he was prescribed cephalexin; he did not camp or hike or do any exertional activity in Colorado. No recent exercise or heavy exertional activities per the patient. He noticed his knee swelling and erythema begin to improve. However, he noticed his fever and abdominal pain worsen. When he returned from Colorado, he decided to come to the ED.

**Figure 1:**
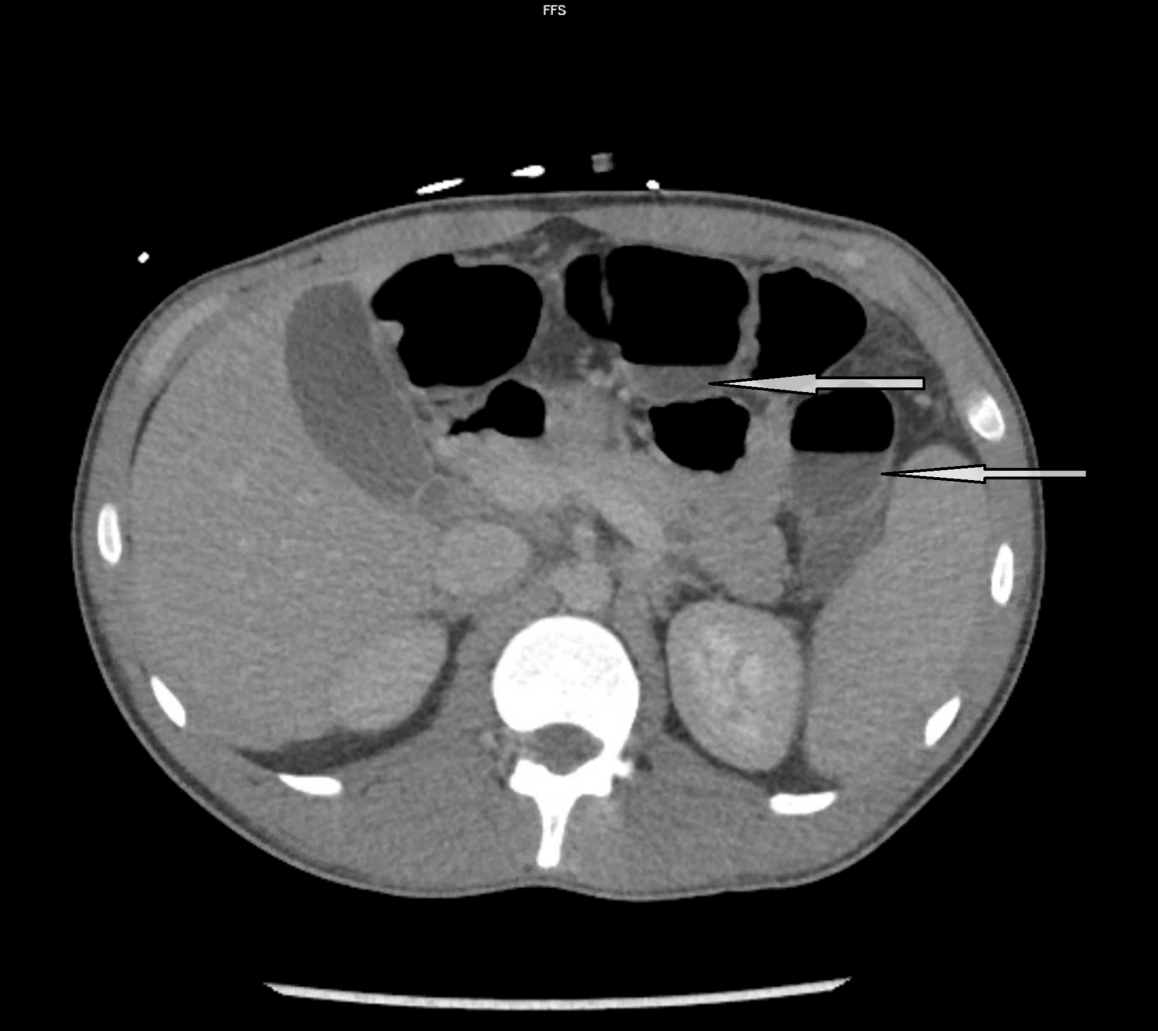
CT scan of the abdomen shows fluid-filled bowels (pointing arrows)

**Figure 2:**
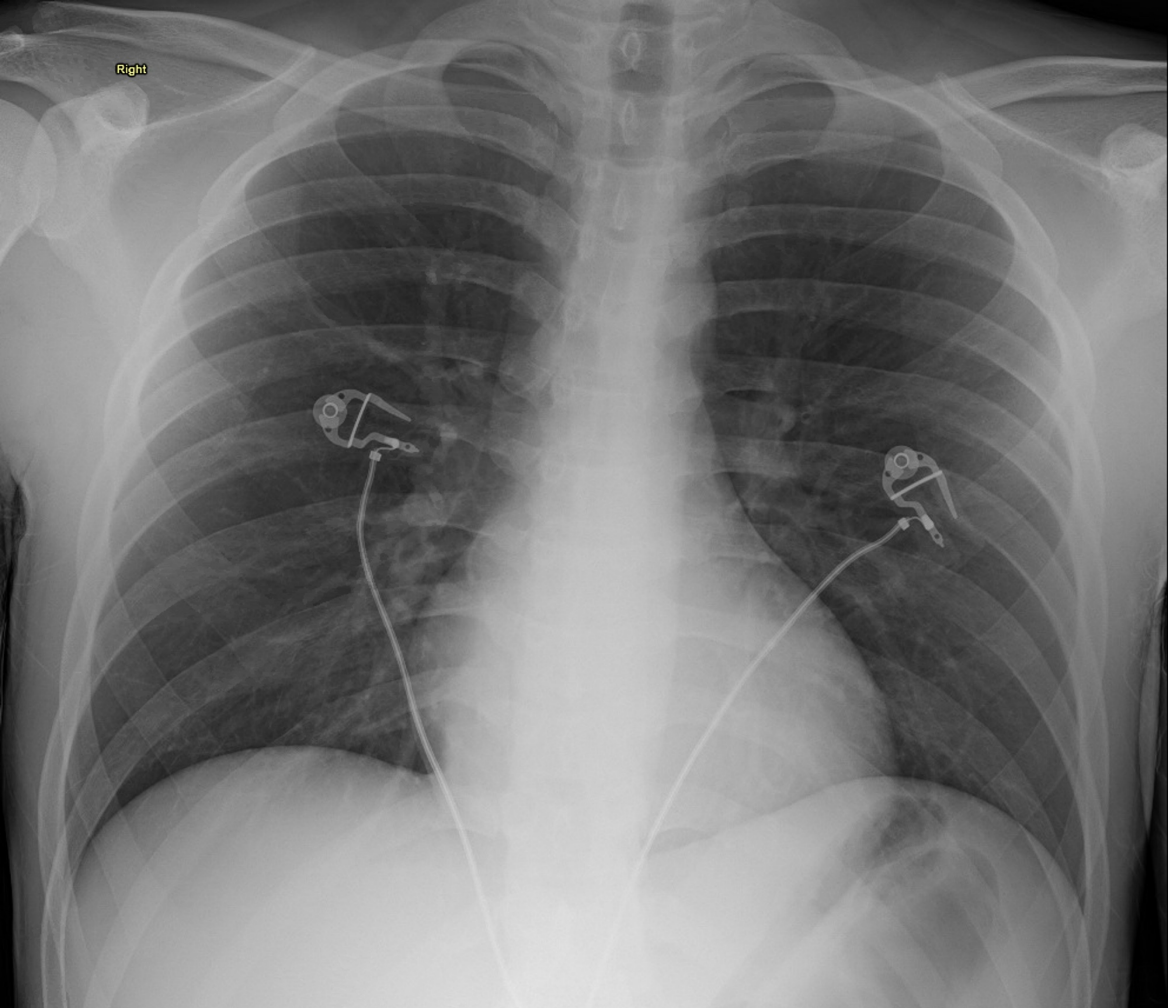
Chest X-ray shows no focal infiltrates, well-inflated lungs

On initial evaluation in the ED the patient was tachycardic at 127 bpm, afebrile (99.5˚F), normotensive (126/73), and had oxygen saturation of 98% on ambient air. On examination, the patient had moderate abdominal tenderness in the left and right lower quadrants. The patient also had a two-week-old 4 centimeter (cm) wide and 2 cm tall left knee wound with erythematous borders and covered with a sanguineous crust which was weeping serous fluid. During a subsequent examination in the ED, the next morning the patient was found to have significant malaise, fever, diaphoresis, a diffusely tender abdomen, and admitted recent COVID-19 exposure. CT abdomen and pelvis revealed a fluid-filled colon but otherwise unremarkable (Figure 1) and view chest X-ray was also unremarkable (Figure 2).

 

**Figure 3:**
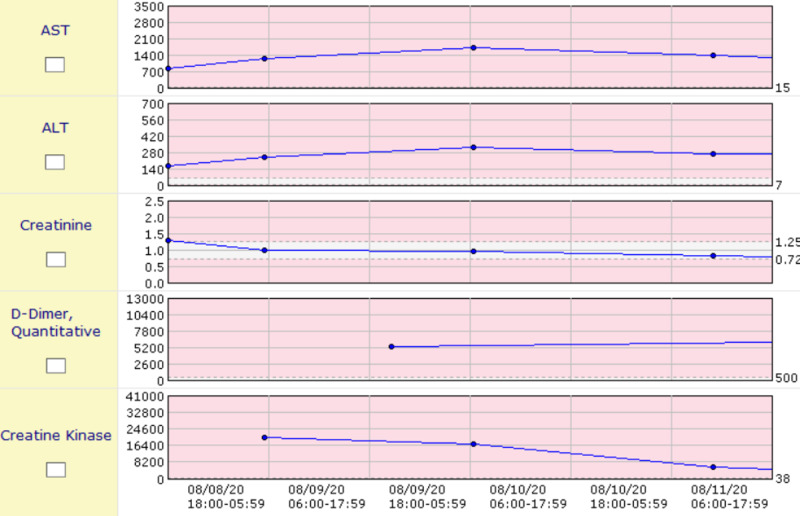
Graphs showing trends of CK levels, AST, ALT, D-dimer, and creatinine ALT, alanine transaminase; AST, aspartate aminotransferase; CK, creatine kinase

**Figure 4:**
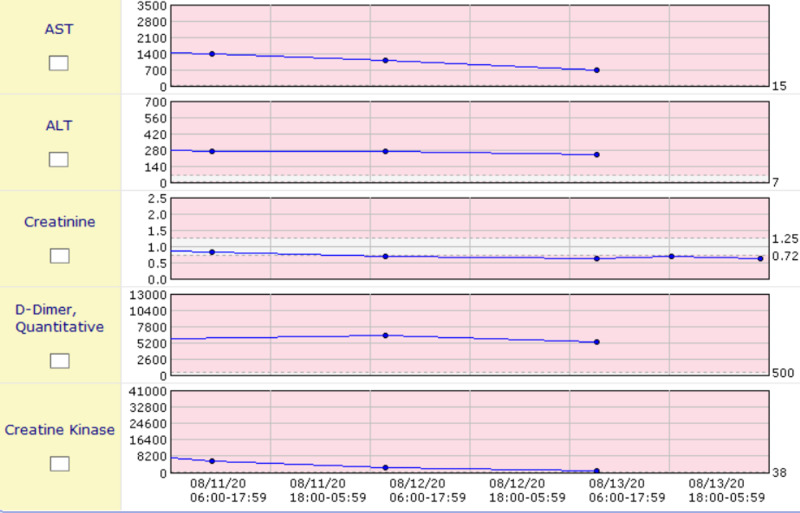
Graphs showing trends of CK levels, AST, ALT, D-dimer, and creatinine ALT, alanine transaminase; AST, aspartate aminotransferase; CK, creatine kinase

Initial lab evaluation revealed white blood cell (WBC) count 19,000 cells per microliter (normal 4,500-11,000), 97% neutrophils, blood urea nitrogen 24 milligrams per deciliter (normal 10-20), creatinine 1.28 milligrams per deciliter (normal 0.84-1.21), sodium 127 milliequivalents per liter (normal 135-145), CO_2 _17 milliequivalents per deciliter (normal 23-29), aspartate aminotransferase (AST) 837 units per liter (normal 10-40), alanine transaminase (ALT) 162 units per liter (normal 29-33), and creatine kinase (CK) 22,000 units per liter (normal 22-198). Of the most importance, the patient was found to be immunoglobulin G (IgG) positive, and polymerase chain reaction (PCR) was positive for COVID-19 infection, and urine drug screen is negative. Due to the patient’s transaminitis, elevated D-dimer, and CK, he was admitted to the COVID-19 floor of the hospital and was started on a heparin drip due to the elevated D-dimer and COVID coagulopathy. The patient had extreme variations in temperature ranging from 103˚F to 94.1˚F for which he was given a Bair Hugger for 12-24 hours until his core body temperature normalized. The patient received testing and was found to be negative for HIV-1 and -2, Clostridium difficile, Haemophilus influenzae, antimitochrondrial antibodies, and antinuclear antibodies. The patient continued to receive aggressive intravenous fluid resuscitation and empiric antibiotics, and his CK levels, creatinine, D-dimer, AST, and ALT continued to normalize, as shown in the graphs (Figures 3, 4).

The patient was insistent on going home regardless of his 103˚F temperature and tachycardia of 116 bpm; however, the patient was instructed to return to the emergency department if his symptoms worsened. 

## Discussion

Rhabdomyolysis is the breakdown of myocytes of skeletal muscle and the release of intracellular contents. Because of this, rhabdomyolysis is characterized by increased levels of CK, myoglobin, potassium, aldolase, lactate dehydrogenase, urate, and ALT. Rhabdomyolysis can be a potentially life-threatening condition due to the complications posed by the release of excess amounts of these intracellular contents. Rhabdomyolysis can cause renal injury when the myoglobin release exceeds the protein-binding capacity, and the excess pigment precipitates in glomeruli. Hyperkalemia, acute renal failure, metabolic acidosis, disseminated intravascular coagulation, compartment syndrome, arrhythmias, and cardiac arrest are potential complications of rhabdomyolysis [6]. 

The common causes of rhabdomyolysis are illicit drugs, alcohol, crush injuries, current medications, seizures, trauma, neuroleptic malignant syndrome, autoimmune diseases, and viral illnesses [6]. Of note, hydroxychloroquine and oseltamivir used in early COVID-19 cases have been reported to be associated with rhabdomyolysis [7]. There have been case reports about the association of rhabdomyolysis with a viral infection such as influenza and severe acute respiratory syndrome [8,9]. According to Fadila et al., about a third of the reported virus-induced rhabdomyolysis are caused by influenza [10]. Recently, there have been a few case reports of rhabdomyolysis and COVID-19. SARS-CoV-2 has been isolated from multiple organs and raises the possibility of the virus infecting striated muscle and potentially leading to muscle breakdown [11].

In our patient, there was no obvious cause for rhabdomyolysis. He did not report any strenuous activity for a few days prior to the presentation, no evidence of trauma, urine drug screen was negative, denied alcohol usage, and the ethanol level at admission was undetectable. After excluding the common causes, we reached a conclusion that rhabdomyolysis in our patient was associated with COVID-19. Our patient’s COVID-19 was diagnosed by the standard real-time reverse transcription-PCR (rRT-PCR) test. Jin and Tong reported the first case of rhabdomyolysis in COVID-19 as a late presentation [12]. They pointed out that focal muscle pain, checking CK, and myoglobin levels are important for detecting rhabdomyolysis in COVID-19 patients. Rhabdomyolysis has also been reported as an initial presentation of COVID-19 [13,14]. The diagnosis of rhabdomyolysis in COVID-19 is difficult due to the overlapping symptoms of generalized muscle weakness and fatigue. Hence, laboratory evaluation supplements history and clinical picture here. CK levels start to rise within 12 hours of the onset of muscle injury and peak at 24-72 hours and return to the baseline over three to five days [15]. The CK level is usually in a few thousand. In our patient, CK level was initially at 20,310 international units/liter (IU/L), which trended down to 851 in four days.

The mechanism of rhabdomyolysis in COVID-19 is not clear, and various mechanisms have been postulated in viral myositis. Immunological mechanisms play an important role in muscle damage by cross-reactivity between the virus and myocytes [16]. Direct invasion of the myocytes like other viral illness can be another mechanism [10,16]. Exaggerated immunological reaction resulting in cytokine storm can lead to muscle damage [17]. T-cell-guided myocyte damage can occur in some viral myositis [18]. 

Rhabdomyolysis is treated with aggressive intravascular volume expansion and addressing the causative agent. Fluid resuscitation should be titrated according to the urine output. Administration of mannitol and bicarbonate may be necessary for certain patients. Electrolyte and metabolic abnormalities should be corrected. Few patients might need dialysis due to persistent acidosis or life-threatening potassium level or oliguric renal failure with fluid overload [6]. Our patient responded well to the aggressive intravenous fluids, and the CK level steadily declined. Overall, the patient responded well to the treatment and supportive care for COVID-19. He improved and was discharged home in a stable condition. 

## Conclusions

Rhabdomyolysis can be an initial extrapulmonary manifestation of COVID-19, with very few cases described in literature so far. It is critical to add CK levels in initial screening labs while evaluating suspected COVID-19 infection with myalgias and generalized weakness, which helps in preventing acute renal failure. Treatment of rhabdomyolysis is with aggressive intravenous fluid hydration, correcting electrolytes, monitoring urine output, serial muscle enzymes, and urine pH monitoring. Exact pathogenesis of COVID-induced rhabdomyolysis is unknown, and prompt treatment will yield good outcome as in our patient. 
